# Supplementary material for: Identification and functional analysis of non-coding regulatory small RNA FenSr3 in Bacillus amyloliquefaciens LPB-18
Source: PeerJ. 2023 May 15;11:e15236. doi: 10.7717/peerj.15236 (PMC10194069; doi:10.7717/peerj.15236)
Supplement: Supplemental Information 4 [file peerj-11-15236-s004.zip › KO/CK-vs-T1_map/map02020.html]

KEGG PATHWAY: Two-component system - Reference pathway


|  |  |
| --- | --- |
| **Two-component system - Reference pathway** |  |

[
Pathway menu
| Organism menu
| Pathway entry
| Show description
| User data mapping
]

Two-component signal transduction systems enable bacteria to sense, respond, and adapt to changes in their environment or in their intracellular state. Each two-component system consists of a sensor protein-histidine kinase (HK) and a response regulator (RR). In the prototypical two-component pathway, the sensor HK phosphorylates its own conserved His residue in response to a signal(s) in the environment. Subsequently, the phosphoryl group of HK is transferred onto a specific Asp residue on the RR. The activated RR can then effect changes in cellular physiology, often by regulating gene expression. Two-component pathways thus often enable cells to sense and respond to stimuli by inducing changes in transcription.

|  |  |  |
| --- | --- | --- |
| Reference pathway | 184% 150% 122% 100% 82% 67% 55% | 图片下载 |
